# The Confounder-Mediator Dilemma: Should We Control for Obesity to Estimate the Effect of Perfluoroalkyl Substances on Health Outcomes?

**DOI:** 10.3390/toxics8040125

**Published:** 2020-12-20

**Authors:** Kosuke Inoue, Atsushi Goto, Takehiro Sugiyama, Cecilia Høst Ramlau-Hansen, Zeyan Liew

**Affiliations:** 1 Department of Epidemiology, Fielding School of Public Health, University of California, Los Angeles, CA 90095, USA; 2 Department of Health Data Science, Graduate School of Data Science, Yokohama City University, Yokohama 236-0027, Japan; agoto@yokohama-cu.ac.jp; 3 Diabetes and Metabolism Information Center, National Center for Global Health and Medicine, Research Institute, Tokyo 162-8655, Japan; tsugiyama-tky@umin.ac.jp; 4 National Center for Global Health and Medicine, Institute for Global Health Policy Research, Bureau of International Health Cooperation, Tokyo 162-8655, Japan; 5 Department of Health Services Research, Faculty of Medicine, University of Tsukuba, Ibaraki 305-8575, Japan; 6 Research Unit for Epidemiology, Department of Public Health, Aarhus University, 8000 Aarhus, Denmark; chrh@ph.au.dk; 7 Department of Environmental Health Sciences, Yale School of Public Health, New Haven, CT 06510, USA; zeyan.liew@yale.edu; 8 Yale Center for Perinatal, Pediatric, and Environmental Epidemiology, Yale School of Public Health, New Haven, CT 06510, USA

**Keywords:** confounder, mediator, perfluoroalkyl substances, body mass index, Danish National Birth Cohort, National Health and Nutrition Examination Survey

## Abstract

Confounding adjustment is important for observational studies to derive valid effect estimates for inference. Despite the theoretical advancement of confounding selection procedure, it is often challenging to distinguish between confounders and mediators due to the lack of information about the time-ordering and latency of each variable in the data. This is also the case for the studies of perfluoroalkyl substances (PFAS), a group of synthetic chemicals used in industry and consumer products that are persistent and have endocrine-disrupting properties on health outcomes. In this article, we used directed acyclic graphs to describe potential biases introduced by adjusting for or stratifying by the measure of obesity as an intermediate variable in PFAS exposure analyses. We compared results with or without adjusting for body mass index in two cross-sectional data analyses: (1) PFAS levels and maternal thyroid function during early pregnancy using the Danish National Birth Cohort and (2) PFAS levels and cardiovascular disease in adults using the National Health and Nutrition Examination Survey. In these examples, we showed that the potential heterogeneity observed in stratified analyses by overweight or obese status needs to be interpreted cautiously considering collider stratification bias. This article highlights the complexity of seemingly simple adjustment or stratification analyses, and the need for careful consideration of the confounding and/or mediating role of obesity in PFAS studies.

## 1. Introduction

Observational studies have played an important role in making inferences about the impact of environmental exposure on health because experimental studies are often unfeasible or unethical to perform. Confounding bias is a major concern that may threaten the validity of observational studies [1]. To reduce confounding, one might think that controlling for as many variables as possible in the model is preferable, but such an approach sometimes introduces bias and reduces statistical efficiency [1,2]. For instance, the total effect of the exposure on health outcomes may be underestimated if an intermediate variable that lies within the causal pathway between the exposure and outcome is included in the statistical model, and, therefore, the estimated (total) effect would fail to capture the specific effect mediated through that variable. In addition, if there are unmeasured confounders between the intermediate variable and the outcome, adjusting for such an intermediate variable could introduce “collider bias” [3]. Some well-known examples of such bias include the “birth weight paradox” [4], where maternal smoking became protective on infant mortality risk conditioning on low birth weight status; or the “obesity paradox” [5] where favorable health risks are observed among obese individuals, conditional on surviving the study follow-up period. These examples have emphasized the potential harmfulness of conditioning or stratifying on intermediate variables affected by the exposure in statistical analyses. However, it is often challenging to determine whether variables are confounders or mediators when the time ordering of the study variables in a dataset is unclear.

Perfluoroalkyl substances (PFAS), a group of man-made fluorine-containing chemicals, has received attention over the past decade, and potential adverse health effect of human exposure to these extremely widespread and persistent compounds have been studied [6]. Although the production of some PFAS, such as perfluorooctane sulfonate (PFOS) and perfluorooctanoic acid (PFOA), has been reduced in Europe and the U.S. since 2000 [7], these compounds are still widely detected [8,9], and several other types of PFAS are reported to be increasing in use [8]. Research has demonstrated that several types of PFAS have endocrine-disturbing properties and can affect steroids and thyroid function [10] as well as cholesterols levels [11,12], leading to cardiovascular disease (CVD) [13]. These previous studies have adjusted for obesity, which is one of the metabolic disorders that has been associated with PFAS in adulthood during reproductive age and beyond [14,15,16]. However, nearly all of the studies were performed using cross-sectional data [10], and therefore, a possible bidirectional relationship between PFAS and obesity makes it difficult to determine whether obesity should be treated as a mediator or a confounder in the statistical analysis, especially if obesity is measured at the same time as PFAS assessment. Moreover, the biological half-life for common PFAS compounds is in the magnitude of years. One measurement of both PFAS and obesity might represent cumulative or chronic status years before data collection.

The goal of this study is to use PFAS research as examples to illustrate how obesity can act as either a mediator or a confounder in studies of environmental exposure and health. We used causal diagrams or directed acyclic graphs (DAGs) to explain the confounding mediator dilemma of defining the role of obesity in PFAS studies. Applying DAGs is useful to guide analysis that aims to estimate the (total) causal effect of the exposure on the outcome, by identifying a sufficient set of variables that can close all confounding paths while avoiding controlling for any mediators and colliders [17,18,19]. As an illustration, we compared results with or without controlling for body-mass-index (BMI) in two cross-sectional studies that estimated the associations of PFAS levels with maternal pregnancy thyroid function [20] and with prevalent CVD in adults [13].

## 2. Confounder-Mediator Dilemma

**Figure 1:**
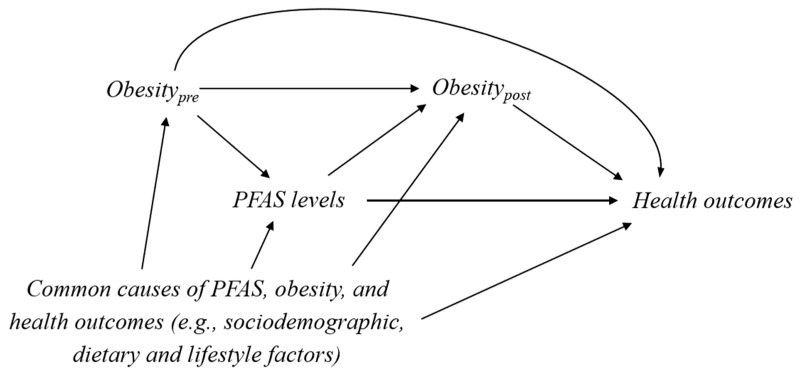
A causal diagram that illustrates the potential causal relationship between PFAS exposure, the measure of obesity, and health outcomes. Obesity_pre_ represents a measure of obesity status before the PFAS measurement and Obesity_post_ represents a measure of obesity after PFAS measurement. Obesity_pre_ affects PFAS levels and health outcomes (i.e., a confounder), while Obesity_post_ is affected by PFAS and affects health outcomes (i.e., a mediator). In this scenario, we should adjust for Obesity_pre_ in addition to other confounding variables such as the common causes of exposure and outcomes to unbiasedly estimate the total effect of PFAS exposure on health outcomes. Adjustment for Obesity_post_ is unnecessary and should be avoided. However, the exact timing for Obesity_pre_ is hard to determine because serum or plasma PFAS levels could represent cumulative exposure. For persistent PFAS compounds, obesity may need to be measured at least a few years ahead of PFAS measurements to be considered as Obesity_pre_.

In general, confounders are variables that are common causes of the exposure and the outcome (i.e., the variable with arrows pointing into both the exposure and the outcome in DAG). More strictly speaking, confounders are variables that are causes of the exposure and related to the outcome, or variables that are causes of the outcome and related to (but not affected by) the exposure [19]. Mediators are variables that lie on the causal pathway from the exposure to the outcome (i.e., the variable with an arrow pointing from the exposure and an arrow pointing into the outcome). Figure 1 shows a DAG that encodes the potential causal relationship between PFAS levels and health outcomes. In this DAG, obesity “before” the PFAS measurement (Obesity_pre_) is a confounder for the causal relationship between PFAS and health outcomes, while obesity “after” the PFAS measurement (Obesity_post_) is a mediator on the causal path from PFAS to health outcomes.

**Figure 2:**
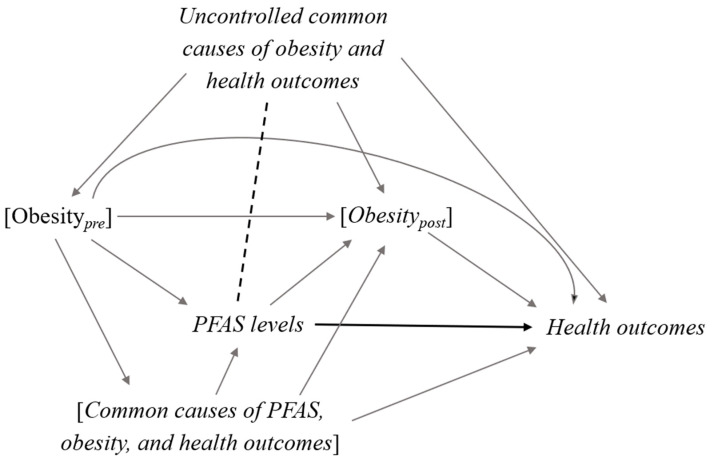
Collider bias due to conditioning on the measure of obesity affected by PFAS when estimating the effect of PFAS exposure on health outcomes. In this DAG, [*variable*] represents the adjustment of the variable. Uncontrolled common causes of obesity and health outcomes are variables that were not included in the model. Measured confounders (common causes between the exposure and outcome) and obesity_pre_ should be adjusted to remove confounding (backdoor) paths between PFAS and health outcomes. However, conditioning on obesity_post_ will (i) close a causal mediating path between PFAS exposure on health outcomes, and (ii) induce a biasing path via uncontrolled common causes of obesity_post_ and health outcomes (i.e., via a spurious association between PFAS and uncontrolled common causes of obesity_post_ and health outcomes as indicated by the dash line in this DAG). The latter is also known as “collider bias”.

To estimate the total effect of the exposure on the outcome, only the confounders, but not the mediators, should be included in statistical analysis. Controlling for intermediate variables can introduce biased estimate mainly for two reasons (Figure 2): (i) failure to include the effect of the exposure on the outcome that is mediated through the intermediate variables, and (ii) introduction of a collider bias, which is the non-existent relationship between the exposure and the outcome through the mediator-outcome confounders created by controlling for the intermediate variables [3,21].

Most epidemiological studies of PFAS at present have information on obesity measured only once at baseline and cannot distinguish between a confounder (obesity_pre_) and a mediator (obesity_post_) due to unknown temporality. Even when we have longitudinal data, it is often challenging to define whether obesity measurement represents the status prior to or post PFAS exposure, because both obesity and PFAS levels represent cumulative or chronic status years before the measurements. In such scenarios, it is important to carefully analyze the data and interpret the results with and without controlling for obesity or BMI (as a proxy measurement of obesity). In the following sections, we illustrate two PFAS studies (of which individual-level data were available or accessible to the authors) using BMI as an example of a variable that could be both a confounder and a mediator between PFAS and health outcomes. In these examples, we focused on PFOS and PFOA, the two most commonly used PFAS, for our exposure of interest, and discussed the results in the following discussion section.

## 3. Example Illustration I: The Association between PFAS and Thyroid Hormones during Pregnancy in the Danish National Birth Cohort

One of the main features of PFAS is its endocrine-disrupting properties, particularly affecting thyroid function [22,23,24]. Whether PFAS exposure during pregnancy affects maternal thyroid function—a critical endocrine function for the fetus neurodevelopment [25,26]—has been actively debated in environmental epidemiology and endocrinology [10,20,27]. A summary of findings about PFAS exposure and maternal thyroid hormones in pregnancy can be found in recent systematic review articles [10,28]. Using a sample of 1,366 pregnancies from the DNBC, a national pregnancy cohort study in Denmark established in 1996–2002 [29], we recently reported possible gestational-week-specific associations between plasma PFAS levels (continuous [per interquartile range increase] or categorical [quartiles with the lowest quartile as a reference] variables) and maternal TSH in early pregnancy [20].

In this work, the results were adjusted for pre-pregnancy BMI, assuming that BMI may affect plasma PFAS levels and maternal thyroid hormones (i.e., obesity_pre_ in Figure 1). This assumption was based on the prior literature of prenatal PFAS exposure research. However, if PFAS measures in early pregnancy represent years of cumulative exposure, pre-pregnancy BMI can potentially be a mediator in the reported PFAS–thyroid association [30,31]. Here, we reevaluated our main findings of thyroid-stimulating hormone [TSH]—a key marker reflecting thyroid function during pregnancy [32], and compared the PFAS-TSH association with or without controlling for pre-pregnancy BMI in the regression model. Moreover, we also performed stratification by maternal overweight status (pre-pregnancy BMI ≥ 25 kg/m^2^). Details of the study design and data collection methods have been described in our previous article [20].

A total of 1366 pregnant women were included in the study. The median (interquartile range) value of PFOS and PFOA measured in maternal plasma collected during GW5 to GW19 (median [interquartile range] = 8 [7,8,9,10]) were 29.5 (22.6–37.7) ng/mL and 4.52 (3.38–5.80) ng/mL, respectively. In multivariable regression analyses, the effect estimates were close to null between prenatal PFOS or PFOA and TSH levels before and after adjusting for pre-pregnancy BMI (Table 1). In an analysis stratified by maternal overweight status, a tendency for positive associations between PFAS, especially for PFOA, and TSH among non-overweight women were observed. However, for overweight women, the effect estimates between PFOS and PFOA levels and TSH went in the negative direction, and for specific exposure quartiles, some estimates excluded the null (Table 2).

## 4. Example Illustration II: The Association between PFAS and Cardiovascular Disease Using the US National Health and Nutrition Examination Survey

A number of studies have suggested that higher PFAS exposure levels are associated with glucose homeostasis and cholesterol levels [11,12,33], which are strong risk factors for CVD. Moreover, a recent cross-sectional study using the NHANES 1999–2000 and 2003–2014 waves (PFAS was not measured in the 2001–2002 wave) showed that the serum PFAS levels were associated with the prevalence of CVD (the self-reported physician diagnosis) in a representative sample of U.S. adults age ≥ 20 years [13]. Previous findings of PFAS exposure and CVD have been summarized in a recent review paper [28]. In the NHANES report [13], BMI of the participants at the time of the survey was included as a confounder (i.e., obesity_pre_ in Figure 1), and thus adjusted for in all regression models. However, if PFAS exposure increases the risk of obesity, BMI could act as a mediator (i.e., obesity_post_ in Figure 1) instead. Whether BMI here represents obesity_pre_ or obesity_post_ in Figure 1 cannot be determined using this cross-sectional data.

Using the publicly assessable data, we re-evaluated the associations between PFAS (continuous [per interquartile range increase] or categorical [quartiles with the lowest quartile as a reference] variables) and prevalence of CVD in NHANES 1999–2000 and 2003–2014 with or without adjusting on participant’s BMI at the time of the study. Moreover, in this U.S. adult sample, we performed stratified analyses classifying participants into obese (current BMI ≥ 30 kg/m^2^) or non-obese (current BMI < 30 kg/m^2^). We used a different cut-off of BMI (i.e., 30 instead of 25 kg/m^2^) between the DNBC cohort and the NHANES cohort because the former included pregnant women in Denmark with a low prevalence of obesity while the latter included all adults aged ≥ 20 years in the US with a high prevalence of obesity [34]. Details of the study sample selection, the measurements of each variable, and the statistical approach are described in Text S1.

Among 7411 US adults included in this complete-case analysis, 571 participants (7.7%) had CVD. The median (interquartile range) values of PFOS and PFOA were 14.1 (7.9–24.4) ng/mL and 3.5 (2.2–5.2) ng/mL, respectively. Similar to the findings reported previously, we found that higher PFOS and PFOA levels were positively associated with the prevalence of CVD and the effect estimates did not change in the model without (Model 1) or with (Model 2) adjustment of current BMI (Table 3). In stratified analyses, a stronger positive association between PFOS or PFOA levels and CVD prevalence was observed for obese adults while the association was null for non-obese participants (Table 4).

## 5. Discussion

This study highlights the complex role of obesity measurement in PFAS exposure and health outcome research. Over-adjustment problems caused by conditioning on intermediate variables require attention in toxicological and environmental epidemiology given that many factors (e.g., medical conditions, biomarkers, and socioeconomic status, etc) are candidates to be potential mediators in the exposure-outcome relationship. Particularly, one measure of obesity can simultaneously act as a confounder and a mediator as well as a “collider” intersecting multiple causal and non-causal pathways, linking PFAS exposure to a health outcome. A naïve adjustment of obesity measurement in a regression model may remove confounding but also poses a risk of blocking the mediating effect of interests. Moreover, conditioning on an intermediate variable between the exposure and the outcome can also risk an induction of collider bias [3,21], which is often being amplified in stratified analyses as seen in the birth weight paradox [4,35] and obesity paradox [5]. We suggest careful consideration of the complex role of obesity in analyses of PFAS exposure on a health outcome, thus results from models adjusting or stratifying on obesity measurement need to be interpreted with caution. Having longitudinal measures of BMI and PFAS levels might help to clarify the time–orders relationship between the exposure and the covariate and appropriate statistical modeling strategies that need to be employed [36,37,38].

Animal studies have suggested that the underlying mechanisms of the PFAS–obesity association might include the activation of the peroxisome proliferator-activated receptors and other transcriptional factors [39,40], the alteration of lipid metabolism [41], and the alteration of energy metabolism and thyroid hormone homeostasis [22,23,42]. These mechanisms indicate that higher PFAS exposure may increase the risk of obesity and possibly other metabolic disorders and CVD. Meanwhile, obesity can also share several common causes with PFAS levels such as dietary and lifestyle factors, thus obesity has been often used as a proxy variable to address lifestyle confounding factors that can be unmeasured or unknown [43,44].

In our illustration examples using the DNBC (PFAS-thyroid association) and the NHANES (PFAS-CVD association), we did not find a substantial change in estimates after adjusting for BMI. In both studies, BMI might not be a strong confounder in the exposure-outcome associations. Particularly for our second example using the NHANES, several variables related to metabolic disorders highly correlated with BMI were already included in the multivariable model. Similarly, BMI might not be a strong mediator in both studies given the very slight changes in effect estimate after adjusting for BMI. However, the potential heterogeneity observed in stratified analyses by overweight or obese status could still be due to collider stratification bias, if there is the presence of other uncontrolled common causes of BMI and the health outcome (i.e., an open biasing path tracing PFAS → [BMI] ← other controlled risk factors → health outcomes in DAG). The direction and magnitude of such bias within strata of a collider can vary and are hard to predict [45,46]. In the NHANES data, measures of PFAS and BMI both can be after the participants had CVD, thus a more direct collider path is possible (i.e., an open biasing path tracing PFAS → [BMI] ← health outcomes in DAG). Pre-pregnancy BMI in the DNBC indicated weight at pre-pregnancy period before PFAS was measured in pregnancy, but PFASs are very persistent, thus one serum measure could represent cumulative exposure years prior to pregnancy. These examples highlight the complexity of seemingly simple adjustment or stratification analyses routinely performed in epidemiological analyses of PFAS.

In this article, we re-evaluated the main findings reported from two recently published articles on PFAS exposure. Our results were meant for illustration and do not reflect the full scope of considerations needed to study the potential causal effect of PFAS on pregnancy thyroid hormones or CVD risk in adults. Our illustrations were based on the two most common types of PFAS and we focused on explaining the potentially complex role of one covariate (i.e., BMI as a measurement of obesity) in analyses. Obesity or overweight measurement was based on self-reported BMI, and stratification analysis was done using a binary classification for simplicity purpose and considering the distribution of BMI in these datasets. Although, how obesity should be considered in causal analyses has been debated in epidemiology [47,48,49], we simply focused on the role of obesity measure as a confounder or a mediator in PFAS studies.

In summary, careful consideration of the confounding and/or the mediating role of obesity is critical for researchers not to misinterpret the data in PFAS studies, particularly when stratifying the study sample by individuals’ obese or overweight status. If the variable is highly likely to be a mediator of the causal pathway of interest, we recommend to refrain from conducting and interpreting the stratified analysis by obesity or overweight, because the stratum-specific effect estimates could be biased. Measuring and adjusting for common causes of PFAS and obesity such as sociodemographic, dietary and lifestyle factors that are less likely to be in the mediating pathways would be recommended to address confounding between PFAS exposure and health outcomes.

## Figures and Tables

**Table 1:** Associations between plasma PFAS levels (ng/mL) and maternal thyroid hormone levels with or without adjustment of pre-pregnancy body mass index (BMI; *n* = 1366).

PFAS	Relative % Difference of TSH (95% CI) ^1^	
	Model 1 ^2^	Model 2 ^3^
**PFOS**		
Per IQR increase	1.06 (0.96, 1.16)	1.04 (0.96, 1.14)
Quartile 1	Ref	Ref
Quartile 2	0.85 (0.68, 1.07)	0.86 (0.69, 1.06)
Quartile 3	0.93 (0.75, 1.16)	0.96 (0.78, 1.17)
Quartile 4	1.03 (0.84, 1.27)	1.01 (0.83, 1.22)
**PFOA**		
Per IQR increase	1.02 (0.94, 1.11)	1.01 (0.93, 1.10)
Quartile 1	Ref	Ref
Quartile 2	0.95 (0.76, 1.19)	0.96 (0.78, 1.19)
Quartile 3	1.01 (0.81, 1.25)	1.02 (0.83, 1.25)
Quartile 4	1.09 (0.86, 1.39)	1.08 (0.86, 1.36)

IQR, interquartile range; TSH, thyroid-stimulating hormone; BMI, body mass index. ^1^ Multivariable linear regression models were employed to estimate the relationships between PFAS levels and ln-TSH. ^2^ Adjusted for maternal age, parental socio-occupational status, parity, maternal smoking, and birth year. ^3^ Adjusted for pre-pregnancy BMI (<18.5, 18.5 to <25, 25 to <30, and ≥30 kg/m^2^) in addition to variables included in Model 1.

**Table 2:** Associations between plasma PFAS levels (ng/mL) and maternal thyroid hormone levels stratified by maternal overweight status indicated by body mass index (BMI).

PFAS	Relative % Difference of TSH (95% CI) ^1^		
	Non-Overweight, BMI < 25 (*n* = 1002)	Overweight, BMI ≥ 25 (*n* = 364)	*P for Interaction* ^2^
**PFOS**			
Per IQR increase	1.08 (0.97, 1.21)	0.94 (0.82, 1.09)	0.19
Quartile 1	Ref	Ref	-
Quartile 2	0.87 (0.67, 1.13)	0.79 (0.56, 1.12)	0.83
Quartile 3	1.05 (0.83, 1.33)	0.64 (0.42, 0.98)	0.15
Quartile 4	1.03 (0.83, 1.28)	0.95 (0.66, 1.35)	0.84
**PFOA**			
Per IQR increase	1.04 (0.94, 1.15)	0.95 (0.82, 1.10)	0.24
Quartile 1	Ref	Ref	-
Quartile 2	1.09 (0.84, 1.42)	0.68 (0.50, 0.92)	0.03
Quartile 3	1.15 (0.90, 1.46)	0.75 (0.53, 1.05)	0.04
Quartile 4	1.12 (0.86, 1.48)	1.02 (0.72, 1.43)	0.52

IQR, interquartile range; TSH, thyroid-stimulating hormone; BMI, body mass index. ^1^ Multivariable linear regression models were employed to estimate the relationships between PFAS levels and ln-TSH adjusting for maternal age, parental socio-occupational status, parity, maternal smoking, and birth year. ^2^ Tests of heterogeneity were also performed by assessing the *p*-value of the interaction term for each PFAS and overweight status in the regression models.

**Table 3:** Associations between serum PFAS levels (ng/mL) and cardiovascular diseases in models with or without adjustment of body mass index (BMI; *n* = 7411).

PFAS	Prevalence Ratio of Cardiovascular Diseases (95% CI)	
	Model 1 ^a^	Model 2 ^b^
**PFOS**		
Per IQR increase	1.06 (1.03, 1.09)	1.06 (1.03, 1.09)
Quartile 1	Ref	Ref
Quartile 2	1.08 (0.76, 1.52)	1.07 (0.76, 1.51)
Quartile 3	1.19 (0.84, 1.67)	1.18 (0.84, 1.67)
Quartile 4	1.18 (0.85, 1.64)	1.19 (0.86, 1.66)
**PFOA**		
Per IQR increase	1.04 (1.01, 1.08)	1.04 (1.01, 1.08)
Quartile 1	Ref	Ref
Quartile 2	1.01 (0.75, 1.36)	1.00 (0.75, 1.34)
Quartile 3	1.14 (0.82, 1.58)	1.14 (0.83, 1.56)
Quartile 4	1.30 (0.99, 1.70)	1.31 (1.00, 1.71)

IQR, interquartile range; eGFR, estimated glomerular filtration rate; HbA1c, hemoglobin A1c; BMI, body mass index. ^a^ Multivariable modified Poisson regression models were employed to estimate the relationships between PFAS levels and CVD prevalence adjusting for age, sex, race/ethnicity, education status, income, marital status, smoking, systolic blood pressure, eGFR, HbA1c, statin prescriptions, and history of cancer. ^b^ Adjusted for BMI (<18.5, 18.5 to <25, 25 to <30, and ≥30 kg/m^2^) in addition to Model 1.

**Table 4:** Associations between serum PFAS levels (ng/mL) and cardiovascular diseases stratified obesity status indicated by body mass index (BMI).

PFAS	Prevalence Ratio of Cardiovascular Diseases (95% CI) ^a^		
	Obese, BMI < 30 (*n* = 4796)	Non-Obese, BMI ≥ 30 (*n* = 2615)	*P for Interaction* ^b^
**PFOS**			
Per IQR increase	1.09 (1.04, 1.14)	1.04 (1.01, 1.09)	0.18
Quartile 1	ref	ref	-
Quartile 2	0.89 (0.58, 1.35)	1.34 (0.78, 2.29)	0.28
Quartile 3	1.01 (0.69, 1.50)	1.51 (0.90, 2.54)	0.29
Quartile 4	1.08 (0.72, 1.60)	1.39 (0.85, 2.27)	0.64
**PFOA**			
Per IQR increase	1.01 (0.95, 1.08)	1.07 (1.04, 1.11)	0.10
Quartile 1	ref	ref	-
Quartile 2	0.77 (0.53, 1.11)	1.38 (0.85, 2.23)	0.04
Quartile 3	1.01 (0.65, 1.55)	1.35 (0.87, 2.09)	0.35
Quartile 4	1.03 (0.73, 1.46)	1.79 (1.15, 2.77)	0.05

IQR, interquartile range; eGFR, estimated glomerular filtration rate; HbA1c, hemoglobin A1c; BMI, body mass index. ^a^ Multivariable modified Poisson regression models were employed to estimate the relationships between PFAS levels and CVD prevalence adjusting for age, sex, race/ethnicity, education status, income, marital status, smoking, systolic blood pressure, eGFR, HbA1c, statin prescriptions, and history of cancer. ^b^ Tests of heterogeneity were also performed by assessing the *p*-value of the interaction term for each PFAS and obesity status in the regression models.

## Supplementary Materials

The following are available online at https://www.mdpi.com/2305-6304/8/4/125/s1, Text S1: Methods in “Example Illustration II” using the NHANES.
