# Supplementary material for: The Confounder-Mediator Dilemma: Should We Control for Obesity to Estimate the Effect of Perfluoroalkyl Substances on Health Outcomes?
Source: Toxics. 2020 Dec 20;8(4):125. doi: 10.3390/toxics8040125 (PMC7766757; doi:10.3390/toxics8040125)
Supplement: Supplementary file 1 [file toxics-08-00125-s001.pdf]

Supplementary Materials: The Confounder-Mediator Dilemma: Should We Control for Obesity to Estimate the Effect of Perfluoroalkyl Substances on Health Outcomes?

Kosuke Inoue, Atsushi Goto, Takehiro Sugiyama, Cecilia Høst Ramlau-Hansen and Zeyan Liew

Text S1: Method in "Example Illustration II" using the NHANES

*Method in "Example Illustration I" using the DNBC can be found in our original article [1].

Data Sources and Study Population

The U.S. National Health and Nutrition Examination Survey (NHANES) is a stratified, multistage probability sample of individuals selected at random from the general population through a complex statistical process [2]. Available data include structured interview data and physical examination results, including urine and/or blood samples. The NHANES study protocols were approved by the National Center for Health Statistics [3].

(NCHS) Institutional Review Board, and all participants provided informed written consent at enrollment. Data are collected continuously but released in two-year cycles. The present study includes data from seven cycles of the continuous NHANES cohort (1999–2000, 2003–2004, 2005-2006, 2007–2008, 2009–2010, 2011–2012, and 2013–2014) that collected data on perfluorooctane sulfonate (PFOS) and perfluorooctanoic acid (PFOA). There were 9,146 participants aged ≥20 years at enrollment for whom PFOS and PFOA were available. We excluded participants who lacked information on the below-mentioned covariates (n = 1,735), resulting in a final analytical cohort of 7411 participants (81%).

Measurements

In NHANES, PFAS were detected and quantified in serum using a modification of online solid-phase extraction coupled to reversed-phase high-performance liquid chromatography-tandem mass spectrometry. In this paper, we focused on PFOS (limits of detection, 0.14 ng/mL) and PFOA (limits of detection, 0.07 ng/mL), two of the most common types of PFAS. Detailed laboratory protocols for the measurement of serum PFAS can be found at https://wwwn.cdc.gov/nchs/data/nhanes/2013-2014/labmethods/PFAS_H_MET.pdf.

Having cardiovascular diseases was based on the self-reported physician diagnosis of congestive heart failure, coronary heart disease, angina pectoris, heart attack, and stroke; i.e., answering yes to the questions "Has a doctor or other health professional ever told you that you have congestive heart failure/coronary heart disease/angina pectoris/heart attack/stroke?"

Demographic variables included respondents' age, sex, race/ethnicity, education status, family income levels, marital status, and smoking status. The previous history of diabetes, hypertension, and cancer was self-reported. Weight and height were measured and used to calculate body mass index (BMI). Systolic blood pressure was measured in the mobile examination center, after resting quietly in a seated position for 5 min. Serum creatinine (Scr) measurements were performed according to the laboratory procedure manual for NHANES, from which an estimated glomerular filtration rate (eGFR, mL/min/1.73 m^2) was calculated. HbA1c was measured using high-performance liquid chromatography. Statin prescription was based on the in-person survey where a surveyor asked participants about all prescription medications used in the past 30 days and examined participants' pill bottles.

Statistical analyses

We employed multivariable modified Poisson regression models adjusting for potential confounders to generate prevalence ratios (PRs) of CVD for PFOS and PFOA concentration (continuous [per interquartile range increase] or categorical [quartiles with the lowest quartile as a reference] variables) [4]. We first adjusted for age (continuous and square transformed), sex (men, women), race/ethnicity (non-Hispanic White, non-Hispanic Black, Mexican-American or others), education status (less than high school, high school or General Education Degree, or more than high school), the poverty income ratio (continuous), marital status (married, single), smoking (never, current, or former), systolic blood pressure (continuous), eGFR (<30, 30 to <60, 60 to <90, and ≥90 mL/min/1.73 m^2), HbA1c (continuous), previous cancer (yes/no), and statin prescription use (yes/no). In Model 2, we additionally adjusted for BMI (<18.5, 18.5 to <25, 25 to <30, and ≥30 kg/m^2) in addition to covariates in Model 1. Then, we performed the stratified analysis by obesity status based on BMI (<30 kg/m^2 vs ≥30 kg/m^2) using Model 1. The P-value for the multiplicative interaction term between PFAS and BMI was calculated. Statistical analyses were conducted using Stata version 15. We selected appropriate sample weights to account for unequal probabilities of selecting NHANES participants, as well as nonresponse of those eligible and approached.

References:

1. Inoue, K.; Ritz, B.; Andersen, S.L.; Ramlau-Hansen, C.H.; Høyer, B.B.; Bech, B.H.; Henriksen, T.B.; Bonefeld-Jørgensen, E.C.; Olsen, J.; Liew, Z. Perfluoroalkyl Substances and Maternal Thyroid Hormones in Early Pregnancy; Findings in the Danish National Birth Cohort. *Environ. Health Perspect.* **2019**, *127*, 117002, doi:10.1289/EHP5482.
2. National Center for Health Statistics, Centers for Disease Control and Prevention National Health and Nutrition Examination Survey. Available online: www.cdc.gov/nchs/nhanes.htm (accessed on 18 December 2020).
3. National Center for Health Statistics, Centers for Disease Control and Prevention NCHS research ethics review board (ERB) approval. Available online: www.cdc.gov/nchs/ nhanes/irba98.htm (accessed on 18 December 2020).
4. Zou, G. A Modified Poisson Regression Approach to Prospective Studies with Binary Data. *Am J Epidemiol* **2004**, *159*, 702–706, doi:10.1093/aje/kwh090.

© 2020 by the authors. Licensee MDPI, Basel, Switzerland. This article is an open access article distributed under the terms and conditions of the Creative Commons Attribution (CC BY) license (http://creativecommons.org/licenses/by/4.0/).
